# Split Ring Resonator-Based Bandstop Filter for Improving Isolation in Compact MIMO Antenna

**DOI:** 10.3390/s21072256

**Published:** 2021-03-24

**Authors:** Hashinur Islam, Saumya Das, Tanweer Ali, Pradeep Kumar, Sourav Dhar, Tanushree Bose

**Affiliations:** 1 Department of Electronics and Communication Engineering, Sikkim Manipal Institute of Technology, Sikkim Manipal University, Sikkim 737136, India; hashinur_201910004@smit.smu.edu.in (H.I.); sourav.d@smit.smu.edu.in (S.D.); tanushree.b@smit.smu.edu.in (T.B.); 2 Department of Information Technology, Sikkim Manipal Institute of Technology, Sikkim Manipal University, Sikkim 737136, India; saumya.das@smit.smu.edu.in; 3 Department of Electronics & Communication, Manipal institute of Technology, Manipal Academy of Higher Education, Manipal, Karnataka 576104, India; 4 Discipline of Electrical, Electronic and Computer Engineering, University of KwaZulu-Natal, Durban 4041, South Africa

**Keywords:** MIMO antenna, decoupling network, SRR-based bandstop filter

## Abstract

The ever-growing expectation for high data rates has led to the introduction of multiple-input multiple-output (MIMO) technologies to wireless connectivity. Such a system requires an MIMO antenna with high isolation. At the same time, the MIMO dimension should not be compromised for achieving high isolation. Thus, isolation techniques that do not allow an increase in dimension need to be fostered for MIMO antenna design. In this paper, a novel low-profile, miniaturized MIMO antenna with high isolation was developed considering a split ring resonator (SRR)-based bandstop filter as a decoupling network. The bandstop filter was designed with a unit cell split ring resonator structure and was deployed between two closely spaced monopole MIMO antenna elements to obtain isolation as high as 39.25 dB at 2.61 GHz. Two open-circuit stub lines were attached with the MIMO feeding network to achieve good impedance matching at resonance frequency. The proposed antenna exhibited a peak gain of 3.8 dBi and radiation efficiency of 84%. It had a low envelop correlation coefficient (ECC < 0.12), high diversity gain (DG > 9.95 dB), low mean effective gain ratio (MEG 1/MEG 2 < 0.05 dB), and low channel capacity loss (CCL < 0.042 bits/s/Hz) at resonance frequency. The overall antenna dimension was restricted to $44\;\mathrm{mm}\;\times 22\;\mathrm{mm}\;\left(0.38\;\lambda_{0}\times 0.19\;\lambda_{0}\right)$ for its easy integration in compact wireless devices.

## 1. Introduction

Due to the introduction of numerous multimedia devices, wireless communication networks today are capable of handling higher data rates than previous systems. These multimedia services demand multielement antennas, such as the multiple-input multiple-output (MIMO) antenna system, which is an effective way to improve channel capacity and, thus, data rate. However, accommodating multiple antennas inside a compact wireless device while maintaining high isolation between antenna elements is very challenging. The influence of surface current results in strong coupling between MIMO antenna elements, as well as between the antenna and ground plane. An M × N MIMO communication system can support data throughput up to K times, where K = min (M, N) of a single-input single-output system for an uncorrelated transmitting and receiving communication channel [1]. Hence, channels need to be uncorrelated to achieve a high data rate. The coupling between antenna elements affects the correlation, thereby lowering the data rate [2]. Hence, for the case of MIMO antenna designing, high isolation is always desired. High isolation can be achieved by increasing the physical spacing between antenna elements. However, it is not possible to increase spacing in the application of compact electronic devices where available space is limited. Alternatively, a decoupling network can reduce the coupling or increase the isolation between multiple antenna elements [3,4,5]. Many researchers presented decoupling networks to diminish the coupling between antenna elements for MIMO antenna design [6,7]. A significant improvement in isolation has been observed with decoupling networks. Therefore, the concept of decoupling networks is an effective way of achieving high isolation in MIMO antennas. However, it is often observed that, with the inclusion of a decoupling structure, the overall dimensions of the MIMO antenna are increased. For example, in [8], a planar dual MIMO antenna with a decoupling network occupied overall dimensions of 60 × 95 mm^2^.

Metamaterial structures have been widely used for developing antennas for different applications such as wearable communication [9], Ultrawideband (UWB) communication [10], terahertz communication [11], Radio Detection and Ranging (RADAR) [12], and pattern reconfigurable systems [13,14]. Metamaterial structures have also been considered as decoupling networks to obtain high isolation in MIMO antennas [15,16]. The split ring resonator (SRR), as a metamaterial decoupling network, has been explored by researchers to enhance isolation. Several articles have shown that, when the SRR is subjected to a magnetic field normal to its plane, it retains the negative permeability property [17]. This property makes the SRR a suitable structure for mutual coupling suppression when positioned between patch antennas [18]. A two-port MIMO antenna with a unit cell SRR metamaterial isolator was reported in [19], where a structure dimensions of 47.5 mm × 40 mm held isolation of 20 dB. In [20], a periodic structure of a metamaterial absorber was created between two port MIMO antenna elements to get isolation up to 43.71 dB with comparatively larger dimensions of 71 mm × 42 mm. However, these large dimensions may not be acceptable for compact wireless devices due to size restrictions.

Bandstop filters have also been used as decoupling networks as they can weaken the coupling current in antenna elements and ground plane [21]. The authors of [22] reported a two-port MIMO structure where a fence-shaped bandstop filter was used to achieve isolation of 25 dB with overall dimensions of 50 mm × 35 mm. The authors of [23] presented an internal multiband MIMO antenna with isolation of more than 15 dB in each of its bands with radiating dimensions of 36 mm × 12 mm. Therefore, the bandstop filtering technique may be a good option for reducing the overall dimensions; however, it is unable to exhibit very high isolation.

A new way to design a decoupling network for antenna isolation with a metamaterial-based bandstop filter was presented in [24]. This process of MIMO antenna design brought a reduction in dimensions, as well as an improvement in isolation. Here a negative-permeability SRR metamaterial-based bandstop filter structure was placed between two ports of MIMO antenna elements to decrease the mutual coupling. The MIMO antenna was able to achieve isolation of 35 dB with dimensions of 45.5 mm × 45.5 mm. The concept of the metamaterial-based bandstop filtering technique can be investigated further to reduce the MIMO dimensions and improve the isolation.

In this research work, a novel attempt was made to reduce the overall dimensions and improve the isolation of an MIMO antenna for 2.6 GHz LTE communication. This band is suitable for providing the required capacity to meet the demand for high data rates from a large number of subscribers in metropolitan cities and other high-traffic areas such as airports and industry belts. The SRR-based negative-permeability metamaterial bandstop filter was used as a decoupling network to design a two-port MIMO antenna of reduced dimensions (44 mm × 22 mm) and with an isolation of 39.25 dB. The SRR-based bandstop filter could weaken the coupling current existing between antenna elements, as well as between the antenna and ground plane. The measured results indicate that the MIMO antenna yielded better gain (3.8 dBi) and fair radiation efficiency (84%). In addition, it displayed acceptable values of diversity parameters such as the envelope correlation coefficient (ECC), diversity gain (DG), mean effective gain (MEG), and channel capacity loss (CCL). The research work in this paper demonstrates major advances with respect to [24] in terms of miniaturization and isolation enhancement. The use of a meandered structure in design inspired the effective current path to be accommodated within a smaller area. Thus, the overall dimensions of the designed MIMO antenna were reduced by 53.24%. Furthermore, there was no analysis of gain, efficiency, source of ECC calculation, DG, MEG, etc. in [24]. When considering a radiator as an MIMO antenna, it is very important to analyze the aforementioned parameters for its practical implementation in a wireless communication system. Thus, these investigations were taken into account in the proposed work.

## 2. Antenna Configuration and Design Process

**Figure 1:**
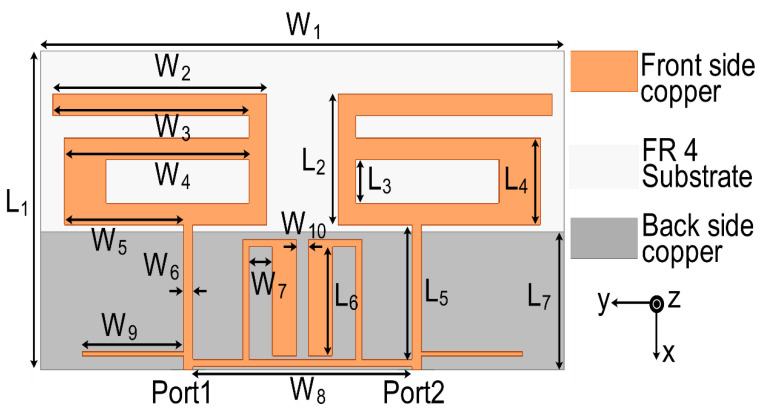
Final configuration of the two-port multiple-input multiple-output (MIMO) antenna.

The final configuration of the proposed MIMO antenna is presented in Figure 1. FR4 material ($\varepsilon_{r}=4.4$, loss tangent tanδ = 0.02, thickness = 1.6 mm) with dimensions of 44 mm × 22 mm was used as the substrate for the antenna. The antenna consisted of two radiation patches, an SRR-based bandstop filter at the top of the dielectric substrate, and a partial ground plane at the bottom of the dielectric structure. Four steps were followed to design the proposed antenna: basic MIMO antenna design, SRR-based bandstop filter design, MIMO antenna with SRR-based bandstop filter design, and stub matching for MIMO antenna. These steps are discussed in this section.

### 2.1. Basic MIMO Antenna Design

**Figure 2:**
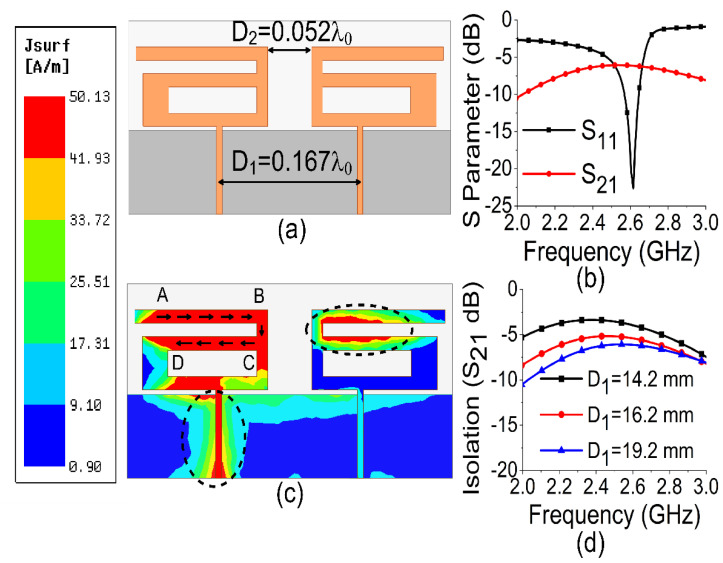
Basic MIMO antenna: (**a**) configuration; (**b**) S parameters; (**c**) current; (**d**) spacing effect between the antennas.

In the first stage of design, a two-port monopole MIMO antenna was configured, as shown in Figure 2a. The simulation results of S parameters confirmed the resonance at 2.61 GHz but showed high mutual coupling between the two ports. This can be observed in Figure 2b with the transmission coefficient (S_21_) and reflection coefficient (S_11_) parameters. The current distribution of the MIMO antenna, shown in Figure 2c, indicated a current path length of 28 mm (ABCD), which caused the structure to resonate at 2.61 GHz following the principle of a quarter wavelength monopole antenna.

**Figure 3:**
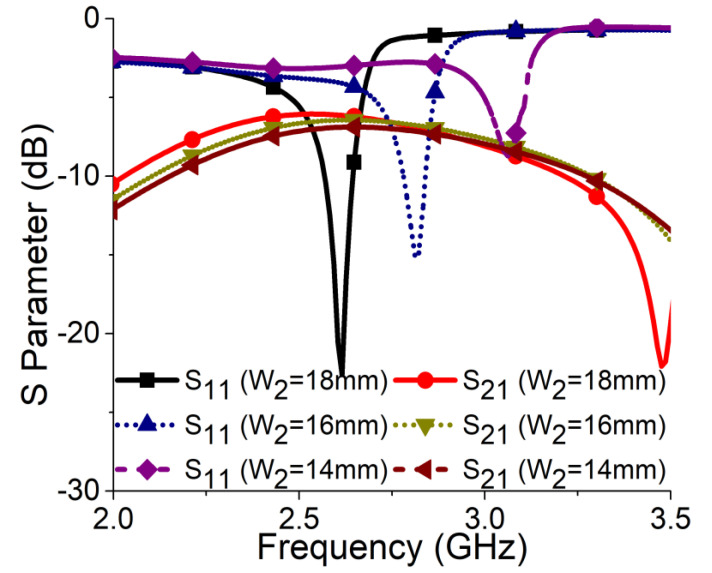
Effect of W_2_ on resonance frequency.

The surface current distribution, shown in Figure 2c, also depicted strong mutual coupling with other antenna elements and the ground plane when port 1 was excited and port 2 was terminated with a 50 Ω matched load. The spacing between antenna elements was optimized in terms of edge-to-edge gap and center-to-center gap. The maximum isolation (7 dB) was obtained with an edge-to-edge gap of 6 mm (0.052 $\lambda_{0}$) and a center-to-center gap of 19.2 mm (0.167 $\lambda_{0}$) at 2.61 GHz, as shown in Figure 2d. Thus, a larger spacing led to better isolation. However, in order to attain a compact dimension, an increase in spacing between antenna elements must be limited. The effect of variations of W2 on scattering (S)-parameters are also illustrated in Figure 3.

### 2.2. SRR-Based Bandstop Filter Design

**Figure 4:**
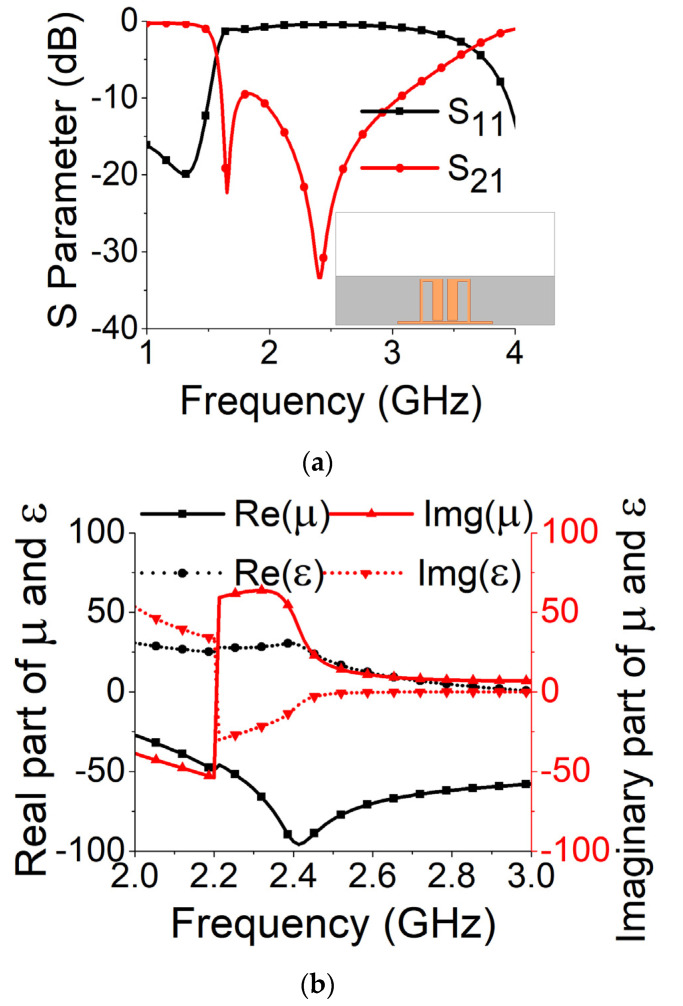
Split ring resonator (SRR)-based bandstop filter: (**a**) S parameters; (**b**) permittivity (ε) and permeability (µ).

To diminish the coupling in the MIMO antenna, an SRR-based bandstop filter was designed on FR4 substrate. It consisted of a unit cell of SRR and feedlines for filter operation. The S parameters of the bandstop filter can be seen in Figure 4a, confirming a band rejection from 1.6 GHz to 3.7 GHz. To ensure the negative permeability of the bandstop filter, the parameter retrieval technique [25] was explored to extract the values of effective permittivity and permeability. The permittivity and permeability values obtained from mathematical analysis are plotted in Figure 4b. At resonance (2.61 GHz), it confirmed the metamaterial property with a negative value of permeability. The negative-permeability SRR induced negative group delay to cancel the coupling currents when placed between two MIMO elements. The negative group delay network acted as a bandstop filter between highly coupled antenna elements.

### 2.3. MIMO Antenna with SRR-Based Bandstop Filter

**Figure 5:**
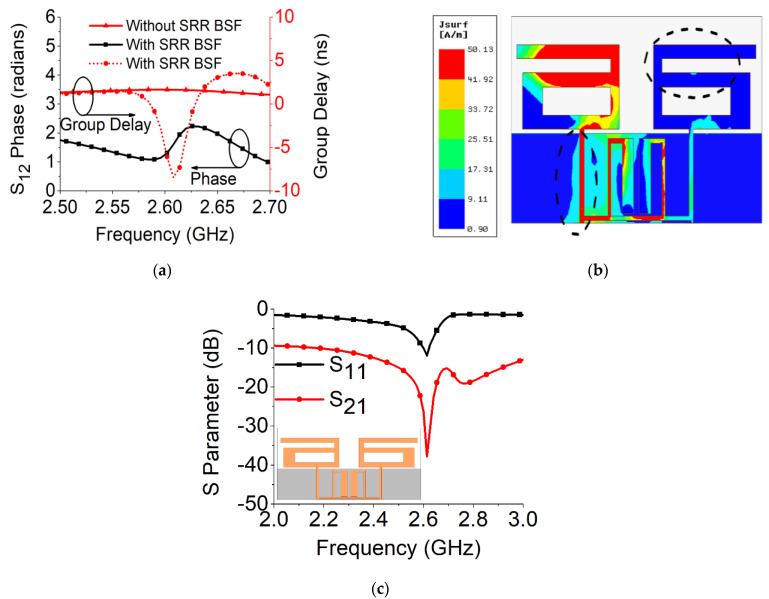
MIMO antenna with SRR-based BSF: (**a**) group delay and phase response; (**b**) current distribution; (**c**) S parameters.

**Figure 6:**
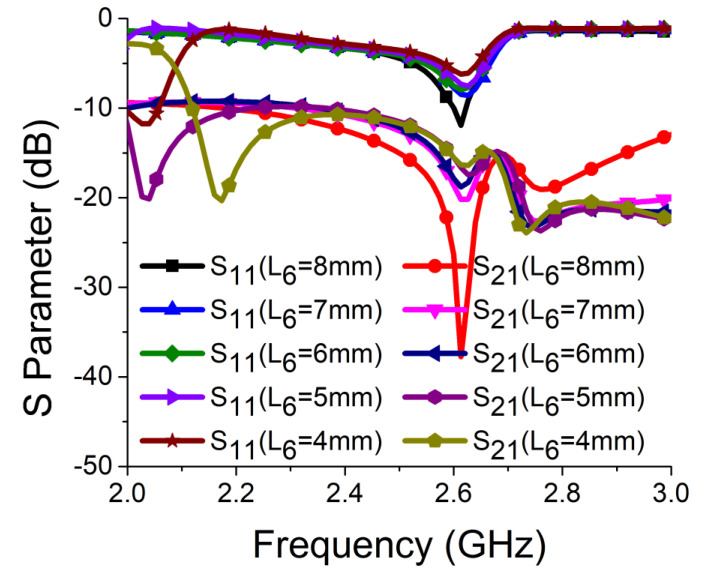
Effect of L_6_ on resonance and isolation.

**Figure 7:**
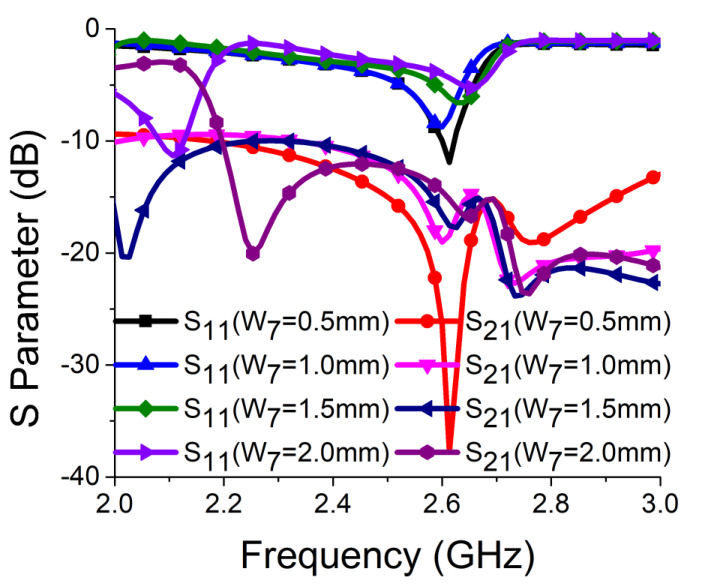
Effect of W_7_ on resonance and isolation.

**Figure 8:**
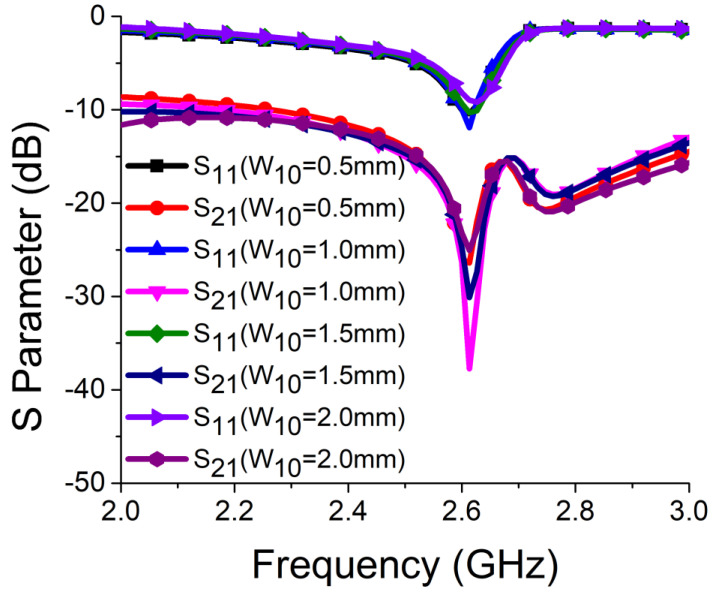
Effect of W_10_ on resonance and isolation.

**Figure 9:**
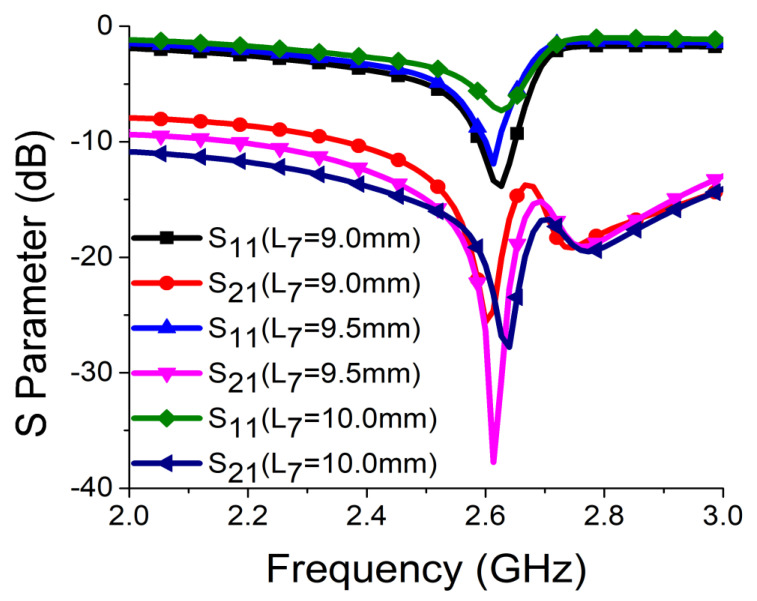
Effect of L_7_ on resonance and isolation.

**Figure 10:**
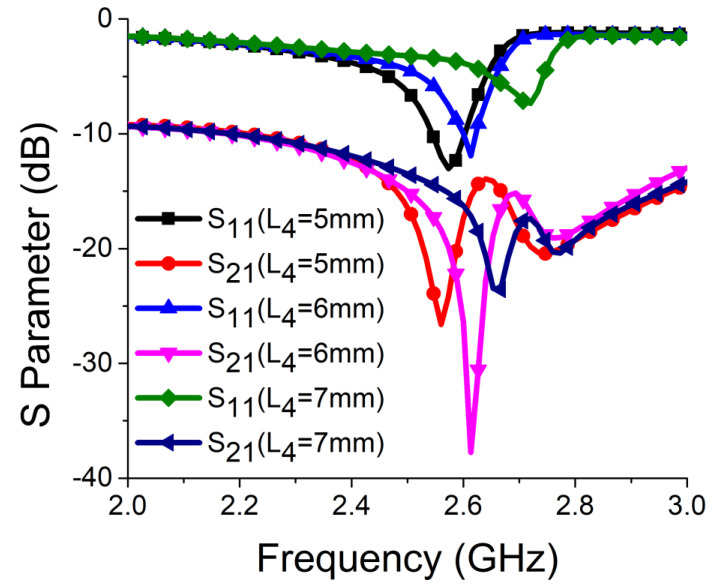
Effect of L_4_ on resonance and isolation.

**Figure 11:**
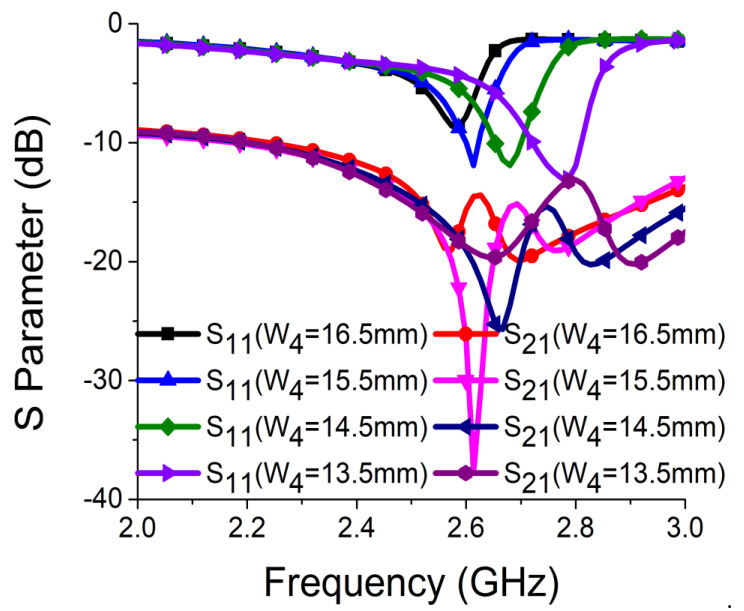
Effect of W_4_ on resonance and isolation.

**Figure 12:**
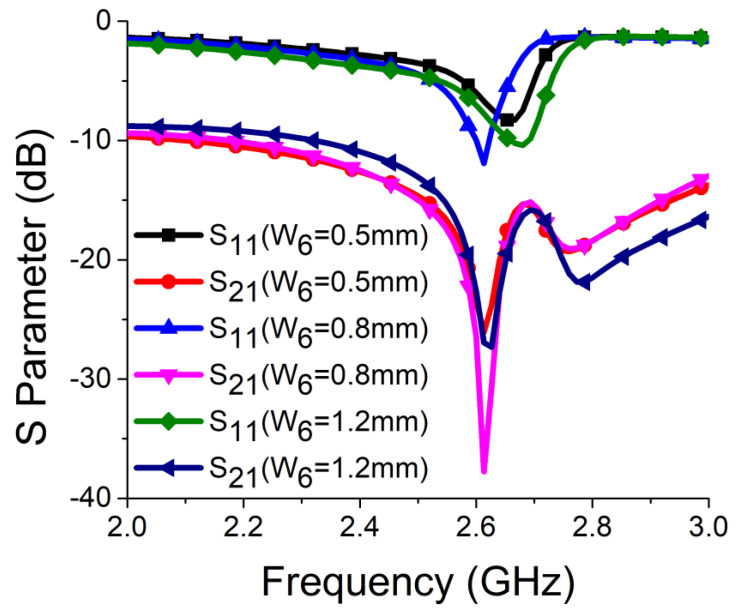
Effect of W_6_ on resonance and isolation.

Strong coupling is primarily due to the identical alignment of the electric fields, which enhances the near-field coupling. The inclusion of the SRR-based bandstop filter in the MIMO structure caused a reversal in the phase response and induced a negative group delay at antenna resonance, as shown in Figure 5a. The change in phase response disrupted the alignment of the electric fields and ensured minimum coupling between the antenna components. This could be confirmed by the current distribution, as shown in Figure 5b. It is evident that much less current was coupled with the other antenna element and the ground plane in the presence of the proposed decoupling network. The S parameter graph in Figure 5c displays a sharp rise in isolation but a decrease in resonance. The degradation of resonance in this structure was due to poor impedance matching. Rigorous parametric analysis was carried out to determine the dimensions of different structural parameters of the antenna structure. L_6_, W_7_, W_10_, L_7_, L_4_, W_4_, and W_6_ played a significant role in increasing the isolation value at 2.61 GHz. The isolation value changed with the change in L_6_ length and achieved optimum resonance and isolation at L_6_ = 8 mm, as shown in Figure 6. The separation between the thick and thin parts of the SRR arm was presented by W_7_. Even a slight variation in W_7_ affected the resonance and isolation significantly. As displayed in Figure 7, the optimum condition at 2.61 GHz was obtained with W_7_ = 0.5 mm. The separation (W_10_) between SRR arms also influenced the resonance and isolation at the desired frequency, as shown in Figure 8. W_10_ = 1 mm yielded the best values of scattering parameters. In this antenna structure, L_7_ represented the length of the partial ground. The partial ground plane included additional reactance in the antenna which could provide better impedance matching. The length of L_7_ was optimized at 9.5 mm to achieve good impedance matching at 2.61 GHz, as shown in Figure 9. An increase or decrease in L_7_ length caused a deviation in resonance frequency. A change in current path length (L_4_ and W_4_) also caused a deviation in resonance frequency and affected the isolation. The values of L_4_ and W_4_ were determined as 6 mm and 15.5 mm, respectively, to optimize the resonance and isolation at 2.61 GHz, as presented in Figure 10 and Figure 11, respectively. The width of the antenna feed line (W_6_) also influenced the resonating frequency and isolation. The variation in W_6_ length, as shown in Figure 12, depicted its best result at 0.8 mm.

### 2.4. Stub Matching Network for MIMO Antenna

**Figure 13:**
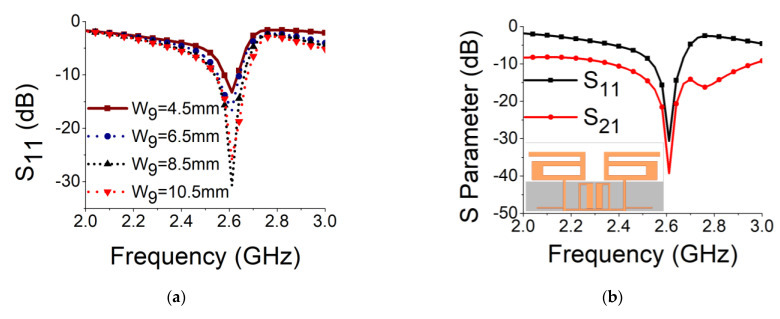

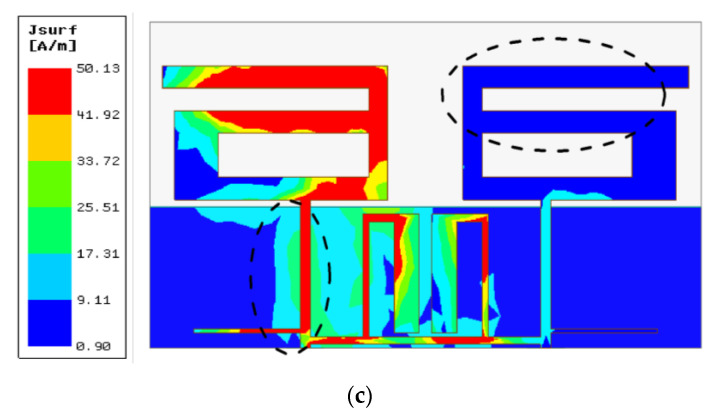
Stub matching network in MIMO antenna: (**a**) effect of W_9_ on impedance matching; (**b**) S parameters of final structures; (**c**) current distribution in final structure.

To bring deep resonance along with high isolation, open-circuit stub lines were attached with MIMO feedlines. Figure 13a shows the improvement in resonance with the increase in stub line length, where the optimal result was achieved at W_9_ = 8.5 mm. The final response of the MIMO antenna is shown in Figure 13b with S parameter values. Figure 13c shows a sharp decrease in coupling current on the other radiator and ground plane in the final structure of the MIMO antenna. The combination of the basic MIMO antenna, SRR-based bandstop filter, and stub lines was able to yield an isolation of 39.25 dB and resonance of −35 dB at 2.61 GHz.

**Figure 14:**
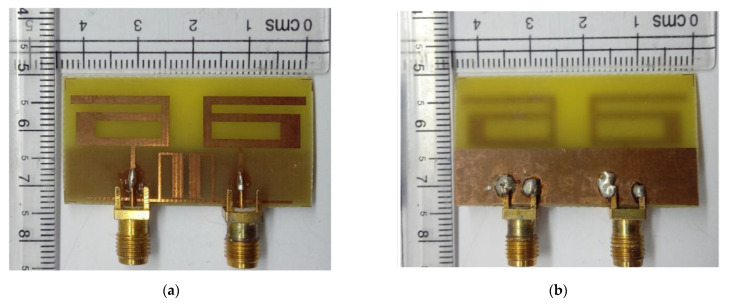
Prototype model of proposed two-port MIMO antenna: (**a**) front view; (**b**) back view.

A simulation was carried out on the High Frequency Structure Simulator (HFSS) platform to optimize the dimensions of design parameters of the proposed MIMO antenna. The optimal design parameters were set as follows: W_1_ = 44, W_2_ = 18, W_3_ = 16.5, W_4_ = 15.5, W_5_ = 10, W_6_ = 0.8, W_7_ = 2, W_8_ = 18.4, W_9_ = 8.5, W_10_ = 1, L_1_ = 22, L_2_ = 9, L_3_ = 3, L_4_ = 6, L_5_ = 9.25, L_6_ = 7.5, and L_7_ = 9.5 (unit: mm). A prototype model of the proposed MIMO structure was developed as shown in Figure 14.

## 3. Results and Discussion

### 3.1. S Parameters

**Figure 15:**
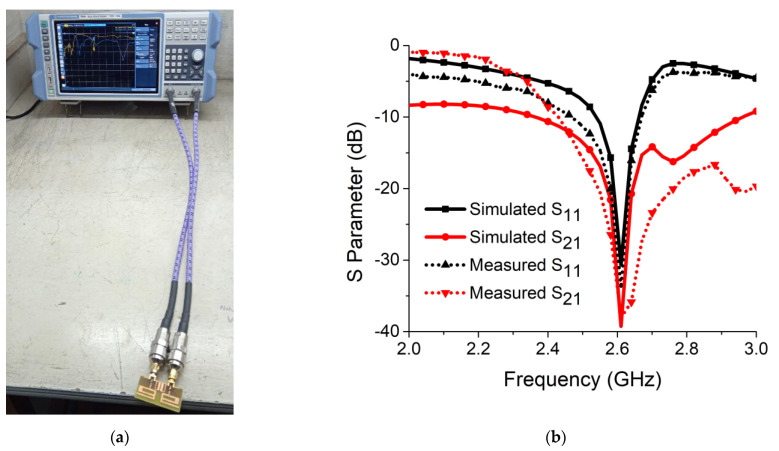
(**a**) Measurement setup of vector network analyzer (VNA); (**b**) simulated and measured results of resonance and isolation.

The simulated results for S parameters were compared with the measured results. The S parameters were measured with a vector network analyzer (VNA), as illustrated in Figure 15a. Simulation findings for isolation (S_21_) and resonance (S_11_) were well aligned with the measured results, as seen in Figure 15b. A maximum isolation of 39.25 dB and S_11_ of −35 dB were obtained at 2.61 GHz. This ensured deep resonance and strong decoupling in the proposed MIMO antenna design. The operational bandwidth obtained at operating frequency was 130 MHz (2.53 GHz–2.66 GHz). The large difference between simulation and measurement for S21 may have been due to errors present in the fabrication process and soldering of the (SubMiniature version A) SMA connector.

### 3.2. Gain, Efficiency, and Radiation Patterns

**Figure 16:**
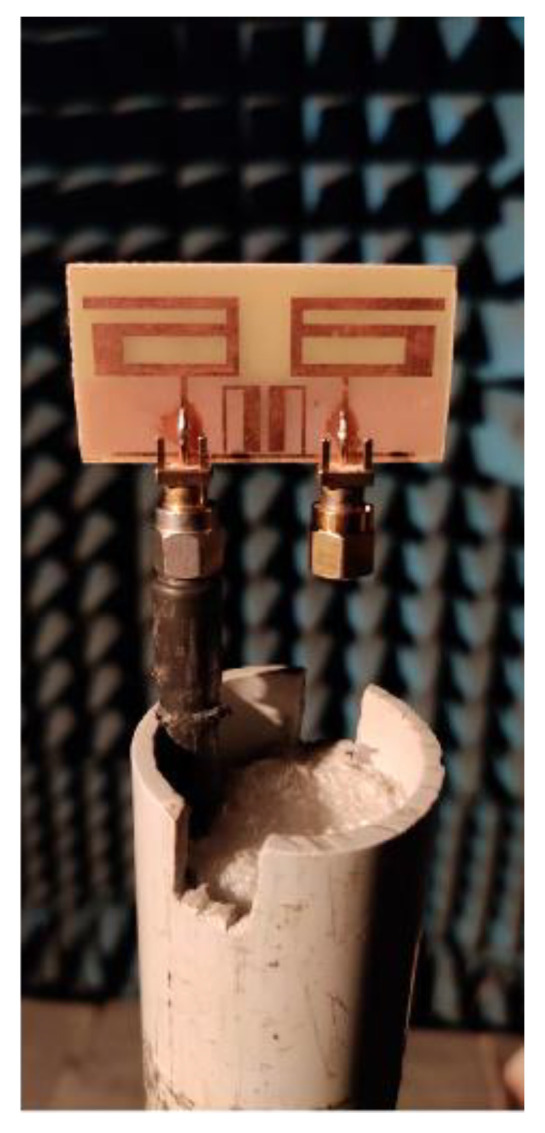
Measurement setup of anechoic chamber to measure radiation characteristics.

**Figure 17:**
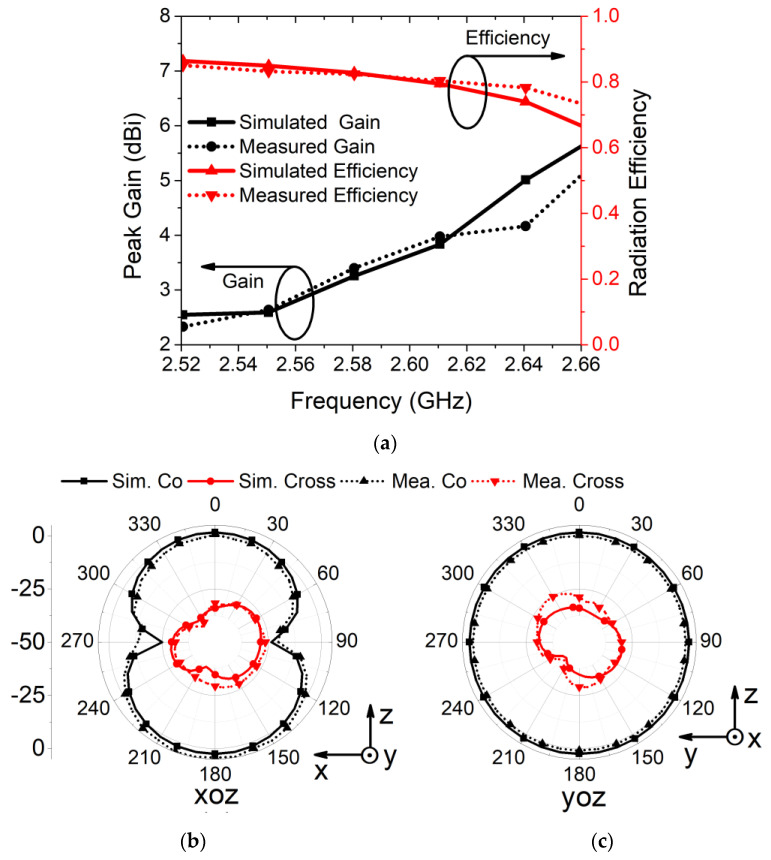
Simulated and measured results of (**a**) peak gain and radiation efficiency, (**b**) radiation pattern of *xoz* plane, and (**c**) radiation pattern of *yoz* plane.

The gain, efficiency, and radiation patterns of the proposed antenna were measured in an anechoic chamber, as illustrated in Figure 16. The gain and radiation efficiency were evaluated at 2.61 GHz for the proposed MIMO antenna structure. It was observed that, at 2.61 GHz, the antenna had a peak gain value of 3.8 dBi and radiation efficiency of 84% in both simulation and measurement, as displayed in Figure 17a. This ensured antenna application for short/medium-range wireless communication. The co-polarization and cross-polarization radiation pattern of the proposed MIMO antenna were observed in the simulation, as well as measurement platform. It was found that the proposed antenna exhibited a bidirectional *xz* plane and an omnidirectional *yz* plane radiation pattern at 2.61 GHz, as displayed in Figure 17b and Figure 17c, respectively. Furthermore, one can notice that the difference between co-polarization and cross-polarization was more than 20 dB in both planes.

### 3.3. Diversity Characteristics

In addition to S parameters and radiation patterns, diversity metrics such as Error Correlation Coefficient (ECC), Diversity Gain (DG), Mean Effective Gain (MEG), Channel Capacity Loss (CCL), and Total Active Reflection Coefficient (TARC) of the proposed antenna were also measured for ensuring the effective utilization of the available environment by the MIMO antenna.

ECC is a measure for describing the isolation or correlation between antenna elements. As the antenna held a good efficiency at 2.61 GHz, ECC could be evaluated following Equation (1) [26].

(1) $$\mathrm{ECC}=\frac{{\left|\iint_{4\pi }\left[\bar{F_{1}}\left(\theta ,\phi \right)\cdot \bar{F_{2}}\left(\theta ,\phi \right)d\Omega \right]\right|}^{2}}{\iint_{4\pi }{\left|\bar{F_{1}}\left(\theta ,\phi \right)\right|}^{2}d\Omega \iint_{4\pi }{\left|\bar{F_{2}}\left(\theta ,\phi \right)\right|}^{2}d\Omega }\;$$

**Figure 18:**
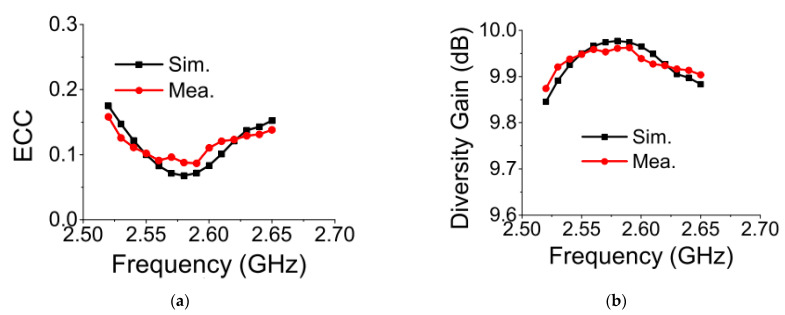
Diversity parameters of proposed MIMO antenna: (**a**) envelope correlation coefficient (ECC); (**b**) diversity gain (DG).

ECC should be 0 ideally, but it is acceptable below 0.5 in a practical environment. As shown in Figure 18a, it is evident that, at 2.61 GHz, ECC for the antenna was obtained as 0.101 and 0.121 from the simulation and measurement, respectively, which is far below the threshold limit. For measuring the ECC, a far-field three-dimensional radiation pattern of the antenna was considered [26].

The value of DG has to be high enough to ensure good quality and reliability of a wireless MIMO system. It should be nearly 10 dB at the operating frequency. The ECC value of the MIMO antenna can be used for calculating DG following Equation (2) [27].

(2) $$\mathrm{DG}=10\times \sqrt{1-\left|\mathrm{ECC}\right|}.$$

Figure 18b shows that the DG value of the proposed MIMO antenna at 2.61 GHz was around 9.95 dB in the simulation and measurement.

To find the TARC value, S parameters can be used for MIMO antennas. The TARC value lies between 0 and 1. The former means that all the available input power is successfully radiated by the antenna [28]. Thus, the TARC value of the MIMO antenna should be near to 0 at the desired frequency. The expression of TARC in terms of S parameters is described in Equation (3) [29].

(3) $$\Gamma_{a}^{t}=\sqrt{\frac{\left(\left({\left|S_{11}+S_{12}e^{j\theta }\right|}^{2}\right)+\left({\left|S_{21}+S_{22}e^{j\theta }\right|}^{2}\right)\right)}{2}}.$$

**Figure 19:**
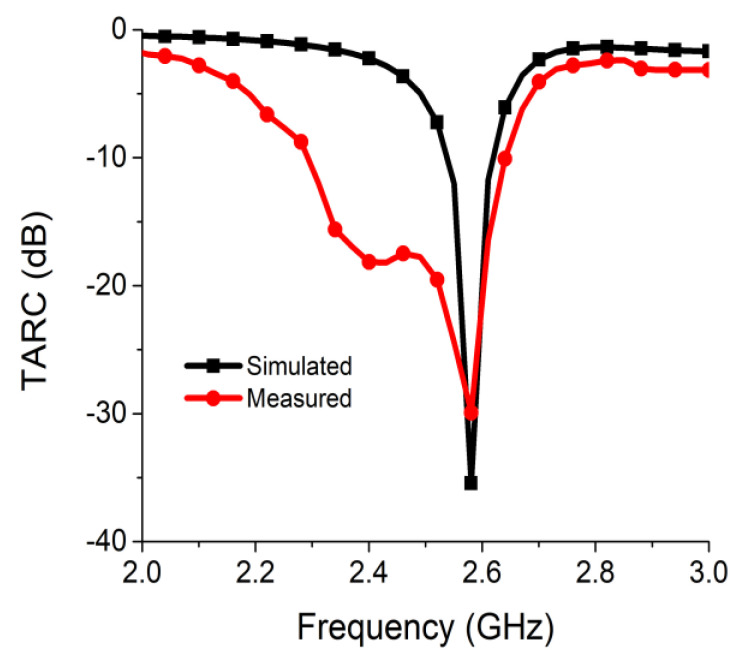
TARC of proposed two-port MIMO antenna.

The evaluated TARC value on the dB scale for the proposed MIMO antenna is displayed in Figure 19. The result ensured satisfactory MIMO operation at the desired frequency.

**Figure 20:**
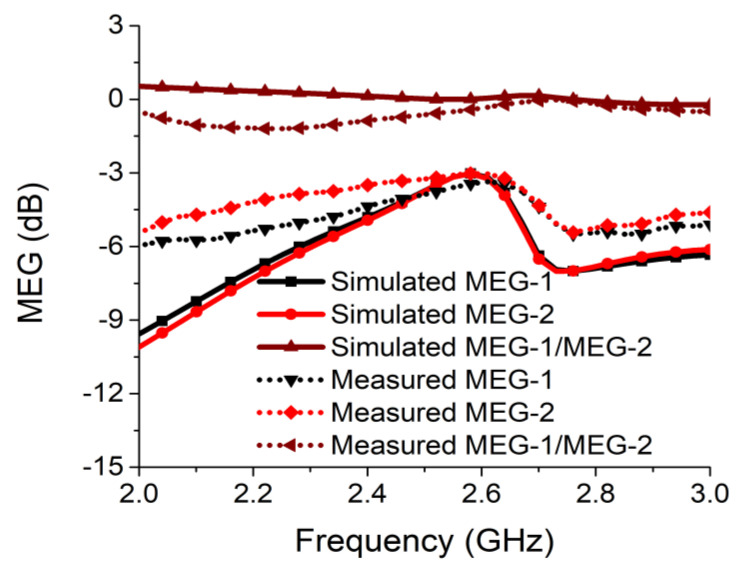
Mean effective gain (MEG) of proposed two-port MIMO antenna.

MEG represents the gain performance of the MIMO radiator, taking the environmental effects into consideration. Figure 20 shows the MEG analysis carried out at port 1 and port 2 following Equations (4) and (5) [30].

(4) $${\mathrm{MEG}}_{1}\;=0.5\left[1-{\left|S_{11}\right|}^{2}-{\left|S_{12}\right|}^{2}\right].$$

(5) $${\mathrm{MEG}}_{2}\;=0.5\left[1-{\left|S_{12}\right|}^{2}-{\left|S_{22}\right|}^{2}\right].$$

The ratio of MEG-1/MEG-2 must be less than 3 dB to ensure a good MIMO design with the same power level at ports. This can be seen in Figure 20, where the ratio was less than 0.05 dB in both simulation and measurement at the desired frequency for the proposed MIMO antenna.

**Figure 21:**
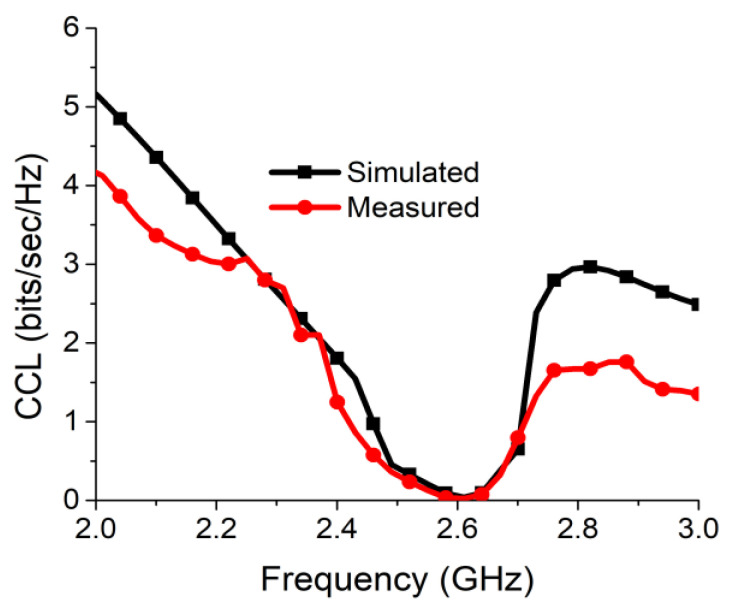
Channel capacity loss (CCL) of proposed antenna two-port MIMO antenna.

The CCL measures the maximum value of channel loss that allows successful message transmission over the communication channel. The CCL should not be more than 0.4 bits/s/Hz for reliable communication. The CCL can be calculated from the formulas mentioned in Equations (6) and (7) [31]. Figure 21 presents the CCL value, which was considerably below 0.042 bits/s/Hz in both simulation and measurement at the desired communication frequency.

(6) $$C_{\mathrm{loss}}=-\log_{2}\left|\varphi^{R}\right|,$$

(7) $$\varphi^{R}=\left[\begin{array}{cc} \varphi_{11} & \varphi_{12}\\ \varphi_{21} & \varphi_{22} \end{array}\right],\;$$

where

$$\varphi_{11}=1-\left({\left|S_{11}\right|}^{2}+{\left|S_{12}\right|}^{2}\right),$$

$$\varphi_{22}=1-\left({\left|S_{22}\right|}^{2}+{\left|S_{21}\right|}^{2}\right),$$

$$\varphi_{12}=-\left(S_{11}^{*}S_{12}+S_{21}^{*}S_{22}\right),$$

$$\varphi_{21}=-\left(S_{22}^{*}S_{21}+S_{12}^{*}S_{11}\right).$$

## 4. Comparative Analysis

A comparative study of a few relevant two-port antennas is presented in Table 1 for the purpose of illustrating the improvement in the proposed antenna. Two-port MIMO antennas with decoupling networks for isolation were taken into consideration. Comparative analysis showed that the proposed antenna had a more compact size than the antenna reported in [8,19,20,22,24]. Furthermore, the proposed antenna exhibited higher isolation at operating frequency than that in [8,19,22,23,24]. The ECC of the proposed MIMO antenna was less than that of the few antennas in the table, but it was well below the required level of 0.5. While isolation in [20] was higher than that of the proposed antenna, the size of the antenna in [20] was much larger than that of the antenna presented in this work.

## 5. Conclusions

In this article, a split ring resonator-based bandstop filter was used as a decoupling network for achieving high isolation and compact dimensions for a two-port MIMO antenna. The bandstop filter was designed with a unit cell split ring resonator structure and was deployed between two closely spaced monopole MIMO antenna elements designed for a 2.6 GHz communication band. To obtain good impedance matching at resonance frequency, two open-circuit stub lines were attached with the MIMO feeding network. The final structure of the MIMO structure exhibited isolation as high as 39.25 dB at 2.61 GHz. Rigorous parametric analysis was carried out to identify the structures responsible for resonance, isolation, and impedance matching. The proposed radiator yielded a maximum gain of 3.8 dBi along with 84% radiation efficiency. It also confirmed near omnidirectional radiation coverage of the surrounding regions. The evaluated values of diversity metrics such as ECC, DG, MEG, CCL, and TARC ensured effective utilization of the available environment by the proposed MIMO antenna.

## Figures and Tables

**Table 1:** Comparison with other decoupling network-based two-port MIMO antennas.

Ref.	Decoupling Technique	Dimension	Resonating Frequency (GHz)	Isolation (dB)	Gain (dBi)	ECC
[8]	Parasitic Elements	0.042 $\lambda_{0}$ × 0.66$\lambda_{0}$	2.1	25	–	–
[22]	Band Stop Filter (BSF)	0.53 $\lambda_{0}$ × 0.37 $\lambda_{0}$	3–11	>25	>3	<0.004
[23]	BSF	0.09 $\lambda_{0}$ × 0.03 $\lambda_{0}$	0.71, 1.92, 2.55	24, 22, 13	−7.5, 3.5, 4.2	–
[24]	Metamaterial (MTM) BSF	45.5 $\lambda_{0}$ × 45.5 $\lambda_{0}$	2.67	35	–	<0.01
[19]	MTM	0.41 $\lambda_{0}$ × 0.41 $\lambda_{0}$	3.5	28	3.2	<0.05
[20]	MTM Absorber	1.30 $\lambda_{0}$ × 0.77 $\lambda_{0}$	5.5	43.71	6.28	<0.05
Prop. work	Split Ring Resonator (SRR) BSF	0.38 $\lambda_{0}$ × 0.19 $\lambda_{0}$	2.61	38	3.8	<0.121

## Data Availability

Not applicable.
